# Dynamic patterns of gene expressional and regulatory variations in cotton heterosis

**DOI:** 10.3389/fpls.2024.1450963

**Published:** 2024-08-06

**Authors:** Chujun Huang, Yu Cheng, Yan Hu, Lei Fang, Zhanfeng Si, Jinwen Chen, Yiwen Cao, Xueying Guan, Tianzhen Zhang

**Affiliations:** ^1^ Zhejiang Provincial Key Laboratory of Crop Genetic Resources, Institute of Crop Science, Plant Precision Breeding Academy, College of Agriculture and Biotechnology, Zhejiang University, Hangzhou, China; ^2^ Hainan Institute of Zhejiang University, Sanya, China

**Keywords:** upland cotton, heterosis, transcriptome sequencing, expression additivity, coexpression network, allele-specific expression

## Abstract

**Purpose:**

Although the application of heterosis has significantly increased crop yield over the past century, the mechanisms underlying this phenomenon still remain obscure. Here, we applied transcriptome sequencing to unravel the impacts of parental expression differences and transcriptomic reprogramming in cotton heterosis.

**Methods:**

A high-quality transcriptomic atlas covering 15 developmental stages and tissues was constructed for XZM2, an elite hybrid of upland cotton (*Gossypium hirsutum* L.), and its parental lines, CRI12 and J8891. This atlas allowed us to identify gene expression differences between the parents and to characterize the transcriptomic reprogramming that occurs in the hybrid.

**Results:**

Our analysis revealed abundant gene expression differences between the parents, with pronounced tissue specificity; a total of 1,112 genes exhibited single-parent expression in at least one tissue. It also illuminated transcriptomic reprogramming in the hybrid XZM2, which included both additive and non-additive expression patterns. Coexpression networks between parents and hybrid constructed via weighted gene coexpression network analysis identified modules closely associated with fiber development. In particular, key regulatory hub genes involved in fiber development showed high-parent dominant or over dominant patterns in the hybrid, potentially driving the emergence of heterosis. Finally, high-depth resequencing data was generated and allele-specific expression patterns examined in the hybrid, enabling the dissection of *cis-* and *trans-*regulation contributions to the observed expression differences.

**Conclusion:**

Parental transcriptional differences and transcriptomic reprogramming in the hybrid, especially the non-additive upregulation of key genes, play an important role in shaping heterosis. Collectively, these findings provide new insights into the molecular basis of heterosis in cotton.

## Introduction

Heterosis refers to the phenomenon where F_1_ hybrids exceed their parents in traits such as growth, adaptability, yield, and quality (Jones, 1917; Duvick, 2005; Hochholdinger and Baldauf, 2018). Heterosis has been leveraged to achieve substantial increases in crop yield, but its underlying genetic basis remains elusive. Numerous studies have proposed models to explain heterosis, such as dominance complementation, single-locus overdominance, and epistasis, along with newer ideas like homoeologous insufficiency (Shull, 1908; East, 1936; Minvielle, 1987; Xiao et al., 1995; Goff, 2011; Xie et al., 2022). Many investigations have focused on identifying heterotic loci by analyzing the parental genomes (Lai et al., 2010; Huang et al., 2015, Huang et al., 2016; Zhang et al., 2016; Gu et al., 2023). Additionally, it has been confirmed that F_1_ hybrids have extensive differences in gene expression in various tissues and developmental stages when compared to their parents (Guo et al., 2006; Shen et al., 2017; Yuan et al., 2017; Zhou et al., 2019; Liu et al., 2022). These differences are likely due to the combined genetic and epigenetic variations from the parental genomes (Chodavarapu et al., 2012; He et al., 2013; Zhou et al., 2019; Luo et al., 2021; Liu et al., 2022). Ultimately, it is clear that transcriptomic reprogramming in hybrids plays a critical role in the formation of heterosis. For instance, a previous study in *Arabidopsis* indicated that genes involved in two different biological pathways exhibited dominance complementation, which promoted biomass heterosis (Liu et al., 2021). In maize, expression complementation driven by genes with single-parent expression (SPE) patterns was also confirmed to contribute to heterosis (Paschold et al., 2014; Marcon et al., 2017). Despite these findings, it remains a significant challenge to integrate expression variations and regulatory networks from both parents and hybrids with the corresponding heterotic performance.

Cotton is a major source of renewable textile fibers worldwide and is also used in the production of oilseeds (Zhang et al., 2015). Xiangzamian 2# (XZM2), being an excellent representative of hybrid cotton and boasting significant yield heterosis, has become the most widely planted hybrid cotton in China. This hybrid was developed by crossing two superior parental lines, CRI12 and J8891. It was first promoted in Hunan Province in 1997, and then expanded to the Yangtze River cotton growing region by 2001, covering an area of more than 1.5 million hectares (Li et al., 1997, Li et al., 2001).

Unraveling the genetic mechanisms behind heterosis in cotton promises to improve the efficiency of hybrid breeding, but remains a critical challenge. Here, we generated transcriptome data for both XZM2 and its parents covering 15 different developmental stages or tissues, creating a relatively complete trio transcriptomic profile. Parental variations in expression and regulation were then systematically characterized, as were expression additivity and allele-specific expression (ASE) in the hybrid. Consensus coexpression networks shared by the hybrid XZM2 and its parents were also constructed. Taken together, these approaches enabled investigation of how parental expression and regulatory variations influence hybrid transcriptomic reprogramming and thereby promote heterosis. The results provide new insights into the molecular foundations of heterosis in cotton.

## Materials and methods

### Plant materials and sampling

The plant materials included the hybrid cotton XZM2 and its parental lines, CRI12 and J8891. The parental seeds used in this study were carefully bred and preserved. To prevent cross-pollination with other varieties, we selected appropriate isolated areas for seed production. Strict field management practices were implemented, including regular weed control and pest and disease management. Seed purity was ensured through morphological evaluation and molecular marker analysis. The F_1_ seeds were obtained by cross-pollinating CRI12 and J8891.

All plants were grown both in the laboratory in Hangzhou, China (120.08°E, 30.30°N) and in the field in Dangtu, China (118.63°E, 31.54°N). Standard field management practices were employed, including fertilization, irrigation, weed control, and pest management, following local conventional protocols. A total of three seedling tissues, including leaves, stems, and roots, were sampled in the laboratory. The remaining twelve tissues were collected in the field, including leaves at budding stage, leaves at blooming stage, ovules at 0–25 DPA, and fibers at 10–25 DPA (**Supplementary Table 1**). To minimize environmental variance, different biological replicates for specific tissues or developmental stages were sampled under identical conditions and at the same time (Stark et al., 2019). In total, 135 samples were collected, covering three genotypes (CRI12, J8891, and XZM2) in 15 different tissues, each with three biological replicates. However, during the RNA-seq library preparation process, one sample failed to meet the minimum sequencing criteria, resulting in two biological replicates for one of the tissues (**Supplementary Table 1**).

### RNA sequencing and data processing

Total RNA was extracted from 135 samples using the Plant RNA Rapid Extraction Kit (Molfarming Biotechnology, No. RK16). RNA-seq was then performed using the Illumina NovaSeq 6000 platform in PE150 mode, resulting in an average of 20–30 million reads per sample. Raw sequencing data from all samples were subjected to quality control using fastp (v0.20.1) (Chen et al., 2018). The clean reads were then aligned to the TM-1 genome (Zhang et al., 2015) using STAR (v2.7.3a) (Dobin et al., 2013) with a key parameter setting of “–sjdbOverhang 149”. Gene expression levels were quantified as transcripts per kilobase of exon model per million mapped reads (TPM) using Stringtie (v2.0) (Pertea et al., 2015).

### PCA and correlation analysis

The RNA-seq count matrix for all 135 samples was filtered to exclude genes with a median read count of zero. The data were then subjected to the variance-stabilizing transformation (VST) function in DESeq2 (v3.18) (Love et al., 2014). PCA was also performed with DESeq2 (v3.18). Pearson correlation coefficients were calculated between all pairs of samples based on the expression matrix of all genes. Two samples were excluded from further analysis due to significantly lower mapping rates or poor correlation between replicate data from the same genotype or tissue (Pearson coefficient < 0.85) (**Supplementary Table 1**) (Schober et al., 2018).

### Identification of parental DEGs

FeatureCounts (v2.0.0) (Liao et al., 2014) was employed to calculate read counts for all genes using the default parameters. DESeq2 (v3.18) (Love et al., 2014) was then used to identify genes that were differentially expressed between CRI12 and J8891 in each developmental stage or tissue. These genes are referred to as DEGs. The criteria for selecting DEGs were set as: adjusted *P* < 0.05 and log_2_(fold change) >= 1.

### Single-parent expression analysis

Based on gene expression levels (TPM) in samples from both CRI12 and J8891, we identified genes that are expressed in only one parent while being silent in the other (Li et al., 2021; Baldauf et al., 2022), referred to as single-parent expressed (SPE) genes. As established in previous research in maize, SPE genes must meet the following two criteria (Zhou et al., 2019):


$$TPM_{A}\leq TPM_{1}$$

$$TPM_{B}\geq 10\times TPM_{A}$$

where TPM_A_ and TPM_B_ indicate the expression of a specific gene in Parent A and Parent B, respectively, and TPM_1_ represents the 1st percentile of expression for all genes under a given condition. For a gene to be considered SPE, its expression in Parent A must be in the bottom 1% of all genes, and it must be expressed at least tenfold higher in Parent B than in Parent A.

### Identification of additive and non-additive expression patterns in the hybrid

Based on gene expression levels in the hybrid, we identified genes with additive and non-additive expression, with the latter category including dominant, over dominant, recessive, and under dominant patterns (Guo et al., 2006; Chen, 2013; Powell et al., 2013). For parental DEGs, all four types of non-additive expression, as well as additive expression, can occur in the F_1_ hybrid. However, for genes that are not differentially expressed between the parents, there are only three possible patterns in the F_1_: additive, over dominant, and under dominant.

For classification of gene expression patterns, Fisher’s exact test was applied to compare expression levels of the hybrid (TPM_F1_) with the mid-parent value (TPM_MPP_), considering genes with *P* < 0.05 as non-additively expressed (Shen et al., 2017). For parental DEGs, those with TPM_F1_ significantly different from TPM_MPP_ and the high-parent value (TPM_high_) but not the low-parent value (TPM_low_) were classified as having “recessive expression”. Conversely, genes that were significantly different from TPM_low_ and TPM_MPP_ but not from TPM_high_ were labeled as having “dominant expression”. For non-DEGs, expression patterns were classified as additive if TPM_F1_ was equal to TPM_MPP_, over dominant if TPM_F1_ exceeded TPM_high_, and under dominant if TPM_F1_ was below TPM_low_ (Shen et al., 2017). All of these classifications were confirmed by Fisher’s exact test using a custom R script with a significance threshold of *P* = 0.05 (**Supplementary Figure 1**).

### Weighted gene coexpression network analysis

The R package WGCNA (v1.72-5) (Langfelder and Horvath, 2008) was used to perform weighted gene coexpression network analysis. Network construction involved two subsets of RNA-seq data, namely samples from ovules at 0–25 DPA and fibers at 10–25 DPA, and incorporated all three genotypes: CRI12, J8891, and XZM2. Genes with an absolute median deviation of expression in the top 75% were retained (Liu et al., 2021). The blockwiseConsensusModules function of WGCNA (v1.72-5) was then invoked to construct a shared network across different genotypes. The main steps in network construction were as follows: 1) calculate the correlation coefficients of expression levels for all genes to create a correlation matrix; 2) apply a soft-thresholding power to the correlation matrix to derive an adjacency matrix; 3) construct a topological overlap matrix to measure the connectivity between gene pairs; 4) perform average linkage hierarchical clustering using the adjacency matrix, resulting in a dendrogram where each branch represents a gene; and 5) use a dynamic cutting method to divide the dendrogram into several large branches, each representing a distinct module. During network construction, the appropriate soft-thresholding power was determined based on the *R^2^* structure of each network, with the requirement that each module contain a minimum of 30 genes. Specifically, in the coexpression network analysis of ovules, we set the soft-thresholding power to 18; whereas in the analysis of fibers, the value was set to 14. Other key parameters included consensusQuantile set to 0, deepSplit level set to 3, DetectCutHeight set to 0.9, and mergeCutHeight set to 0.25.

According to the principles of WGCNA, genes within a module are assumed to be highly coexpressed under specific conditions (such as in a certain developmental stage or tissue) (Langfelder and Horvath, 2008). To examine gene expression patterns within modules shared across genotypes, each module was represented by a module eigengene (ME), computed as the first principal component. Hub genes within a given module are those that connect to multiple genes within the module or are associated with multiple modules. We identified hub genes by calculating the connectivity of each gene to its corresponding module using the module membership (kME) value, defined as the bi-weight mid-correlation between the gene expression and the respective ME (Langfelder and Horvath, 2008). The criteria for selecting hub genes were set as kME ≥ 0.8 and *P* value ≤ 1e-04.

### Gene ontology enrichment analysis

GO enrichment analysis (Ashburner et al., 2000) was conducted using the GO Enrichment function within TBtools-II (v2.086) (Chen et al., 2020), applying a hypergeometric test with FDR correction. The adjusted *P* threshold was set at 0.05.

### Allele-specific expression in the hybrid

High-depth (> 30×) resequencing data for CRI12 and J8891 were mapped to the TM-1 genome (Zhang et al., 2015) to facilitate SNP calling using the HaplotypeCaller and GenotypeGVCFs modules within GATK (v4.1.9.0) (McKenna et al., 2010). High-quality SNPs were then filtered using BCFtools (v1.11) (Danecek et al., 2021), with parameters set as “-m2 -M2 -’F_MISSING > 0.2 | MAC < 3 | MAF < 0.05’”. The parental SNP set obtained was used to characterize ASE patterns in the hybrid.

ASE analysis was performed using phASER (v0.9.9.4) (Castel et al., 2016), with SNPs for detection meeting the following criteria: at least ten reads for each allele, with reads supporting the minor allele making up at least 2% of all reads. Allelic bias in expression was determined for each SNP by a binomial test, with a significance threshold of *P* = 0.05. Similarly, the effect size of ASE regulatory variation (allelic fold change, aFC) was estimated using phASER (v0.9.9.4) (Castel et al., 2016).

### Characterization of *cis* and *trans* regulatory divergence

ASE data from the hybrid XZM2 were utilized to characterize *cis*- and *trans*-regulation. Following the methodology established in previous studies (Bao et al., 2019; Gao et al., 2023), we identified regulatory patterns by comparing the gene expression of different parental alleles in the F_1_ hybrid. A custom R script was employed to determine enrichments of regulatory and additive expression patterns.

## Results

### Transcriptome profiling of various tissues of CRI12, J8891, and their hybrid XZM2

**Figure 1:**
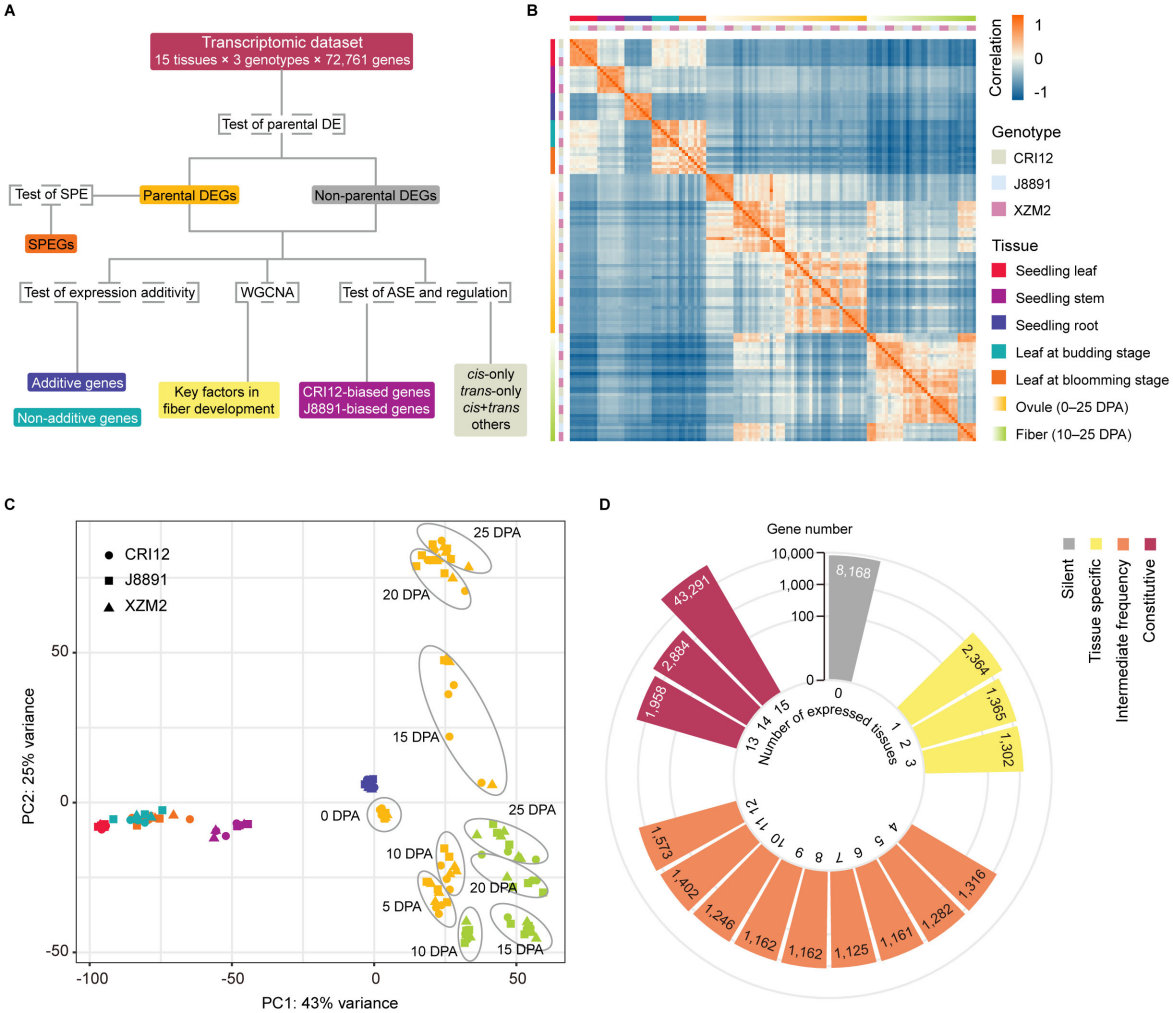
Overview of experimental design and data collected for the study. **(A)** Flowchart summarizing the analyses that were performed. Genes were first divided according to whether they showed evidence of differential expression (DE) between the parents CRI12 and J8891, and then the subset of genes exhibiting single-parent expression (SPE) was identified. Next, additivity of expression was assessed and classified for all genes, and expression data from ovules and fibers were used to construct coexpression networks. Finally, allelic-specific expression (ASE) patterns and *cis*/*trans* regulatory variations were assessed and classified based on a subset of genes that include sequence polymorphisms and are expressed at sufficient levels. **(B)** Heatmap illustrating the pairwise Pearson correlation coefficients (PCCs) of 134 transcriptome profiles from different tissues and genotypes, including three biological replicates. Rectangles on the left and above the heat map indicate sample genotype and tissue. **(C)** PCA visualization of the 134 transcriptome profiles. Shapes and colors indicate sample genotypes and tissues, respectively. **(D)** Counts of expressed genes detected in the 15 investigated tissues. Colors represent the proportion of tissues in which each gene is expressed: grey, not expressed in any tissue (silent); yellow, expressed in less than 20% of tissues (tissue-specific); orange, expressed in 20%–80% of tissues (intermediate); and red, expressed in more than 80% of tissues (constitutive).

XZM2, an elite hybrid of upland cotton, is characterized by its high yield heterosis (**Supplementary Table 1**). It was developed by crossing two elite parents, CRI12 and J8891 (Li et al., 1997, Li et al., 2001). To elucidate the genetic mechanisms of heterosis in XZM2 at the transcriptional level, a systematic transcriptome analysis was performed (**Figure 1A**), providing an opportunity to explore the impacts of expression additivity, allelic regulation, and hybrid-specific expression patterns in shaping heterosis. Transcriptomes were generated for matched tissue samples (note: although referred to as tissues, in some cases these samples represent complex organs comprised of multiple cell types) from numerous developmental stages of both parental lines and the F_1_ hybrid; a total of 135 samples were collected in three biological replicates. The investigated tissues included leaves, stems, and roots at the seedling stage; leaves at the budding and blooming stages; and ovules and fibers at multiple developmental stages (**Supplementary Table 2**). Biological replicates were collected on the same time to minimize the effect of environmental differences on gene expression.

A total of ~1 Tb of sequencing data was generated from 134 samples (excluding one that failed library preparation), with approximately 20–30 million read pairs obtained for each sample. The reads were aligned to the TM-1 reference genome (Zhang et al., 2015), which considers parental variations to reduce mapping bias. Similar unique alignment rates were obtained for the parents and the hybrid, at 88.25%, 89.25%, and 87.34%, respectively (**Supplementary Table 3**). However, two samples were removed from further analysis due to having significantly lower mapping rates or poor correlation with replicates from the same genotype or tissue (**Supplementary Table 3**). The remaining 132 samples showed high reproducibility, with Pearson correlation coefficients between replicates consistently above 0.85 (**Figure 1B**). Clustering by principal component analysis (PCA) reflected sample tissue type, identifying five clusters: leaves, stems, roots, ovules, and fibers (**Figure 1C**). Interestingly, samples of a given tissue type at different developmental stages showed remarkable differences in clustering, indicating great transcriptomic variations between developmental stages (**Figure 1C**).

In the TM-1 genome, a total of 72,761 protein-coding genes are annotated (Zhang et al., 2015). The number of annotated genes expressed in the sequenced tissues varied from 44,698 to 53,701, with an average of 48,695 genes (**Supplementary Table 4**). Interestingly, the hybrid XZM2 showed a slightly higher average number of expressed genes (48,940 genes) compared to its parental lines (48,660 genes for CRI12 and 48,475 genes for J8891) (**Supplementary Table 5**). Of annotated genes, 29,783 were actively expressed in all 15 tissues. A notable number of tissue-specific genes were also identified, with 7,599 genes expressed in only one to three tissues, representing less than 20% of the tissue types examined. Meanwhile, 15,105 genes were expressed in four to 12 tissues. A significant proportion of genes (35,345 in total) showed constitutive expression, being active in more than 80% of the tissues. Finally, 8,168 genes did not show activity in any of the 15 tissues (**Figure 1D**; **Supplementary Table 6**). These could potentially be expressed in other tissues or developmental stages not covered in our research, or might be classified as pseudogenes (Zhou et al., 2019).

### Extensive transcriptomic differences between the parents

**Figure 2:**
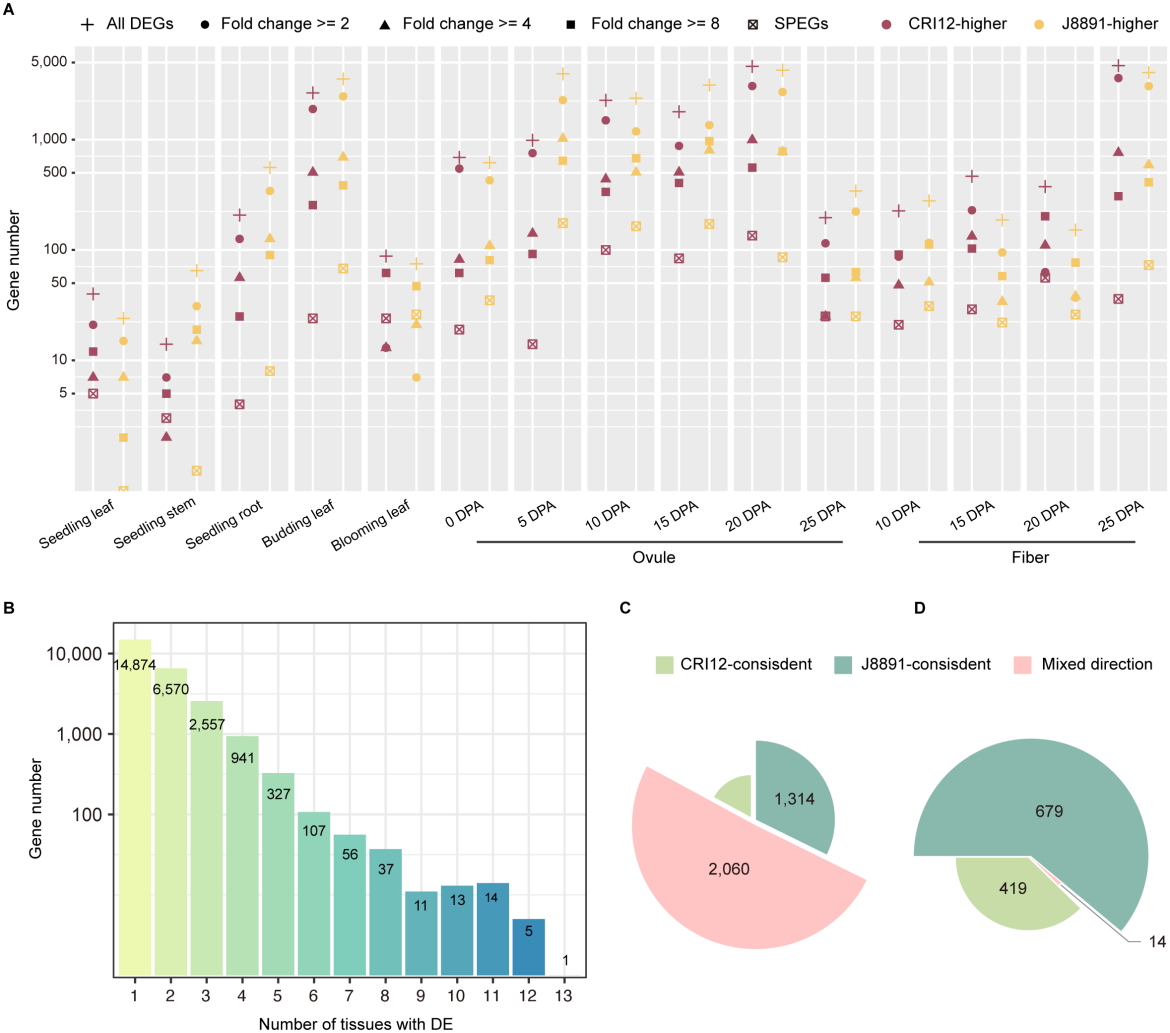
Extensive differences between the two parental transcriptome profiles. **(A)** Plot of the number of parental DEGs for each tissue. Color indicates the genotype with higher expression (purple, CRI12; yellow, J8891). Subsets of genes with >= 2-fold, >= 4-fold, or >= 8-fold changes in expression are indicated by different symbols. The number of genes with SPE patterns is also plotted for each tissue. **(B)** Plot of the number of DEGs detected in 1–13 tissues. The x-axis indicates the number of tissues with the DE pattern. The y-axis indicates the number of DEGs. **(C, D)** Plots illustrating consistency of the parent of higher expression for DEGs **(C)** and SPEGs **(D)** that show DE/SPE in at least two tissues. Light green, CRI12-consistent; deep green, J8891-consistent; pink, mixed direction.

Transcriptome differences between the parental lines, CRI12 and J8891, were characterized by differential expression analysis (**Figure 1A**). A total of 25,513 genes exhibited differential expression in at least one tissue examined. At the individual tissue level, the number of differentially expressed genes (DEGs) varied significantly, ranging from 64 to 8,864 (**Supplementary Table 7**). In eight out of 15 tissues, a greater number of genes were expressed at higher levels in J8891; these tissues included stems and roots at the seedling stage, leaves at the budding stage, ovules at 5, 10, 15, and 25 days post-anthesis (DPA), and fibers at 10 DPA (**Figure 2A**). Conversely, in the remaining seven tissues, CRI12 had a greater number of higher-expressed genes (**Figure 2A**). Examination of the degree of expression difference in these parental DEGs revealed that in the various tissues, a substantial proportion of DEGs (23.70%–83.73%) showed substantial expression changes between the parents (> 4-fold), while another subset of DEGs exhibited moderate changes (**Figure 2A**; **Supplementary Table 8**). Concerning tissue-specificity of DEGs, the majority (21,444, 84.05%) were differentially expressed only in a limited number of tissues (<= 2) (**Figure 2B**; **Supplementary Table 9**). Among the 4,069 genes that demonstrated differential expression in multiple tissues (>= 3), 695 (17.08%) and 1,314 (32.29%) were consistently highly expressed in CRI12 and J8891, respectively. The remaining 2,060 (50.63%) DEGs were mixed in terms of which parent showed higher expression, with some tissues having higher expression in CRI12 and others in J8891 (**Figure 2C**). It is important to note that these directional switches were not merely due to minor expression changes. Among the 4,069 DEGs with mixed directions, 1,525 (37.38%) were observed to have differential expression of more than 4-fold in at least one tissue. Collectively, these results indicated that not only is there a broad range of transcriptomic differences between the parents, but the patterns of differential expression are highly tissue-specific.

Single-parent expressed genes (SPEGs) represent extreme cases of differential expression, as they are typically expressed in only one parent (TPM >= 1) while being completely silent in the other (TPM < 0.1). Previous studies have suggested that SPEGs are important in the formation of heterosis (Marcon et al., 2017; Li et al., 2021; Baldauf et al., 2022). Consequently, we examined SPE patterns between CRI12 and J8891. A total of 1,112 genes were observed to exhibit SPE in at least one tissue, with the number of SPEGs varying from four to 264 across different tissues (**Figure 2A**; **Supplementary Table 10**). Compared to DEGs, SPEGs exhibited a higher degree of tissue specificity: the vast majority (977, 87.86%) displayed SPE only in a single tissue. J8891 possessed slightly more SPE genes, at 679 to the 419 of CRI12. A small subset of SPEGs (135 genes, 12.14%) exhibited SPE in two or more tissues simultaneously. Among these multi-tissue SPEGs, a consistent expression direction was common, with only 14 showing different parent-specificity across tissues (**Figure 2D**).

### Transcriptomic reprogramming in the hybrid XZM2

Transcriptional reprogramming in hybrids relative to their parental lines has been confirmed prevalent in multiple developmental stages and tissues, laying the foundation for heterosis (Liu et al., 2022). The availability of RNA-seq data from XZM2 F_1_ individuals provided an opportunity to characterize the transcriptional reprogramming landscape in this hybrid (**Figure 1A**). In hybrids, gene expression patterns can be categorized into two main types: additive and non-additive. Additive expression refers to instances where the expression level in the hybrid is comparable to the average expression of the parents. In contrast, non-additive expression is observed when expression level in the hybrid deviates significantly from the parental average. Non-additive expression can be further divided into pattern categories spanning the range of expression levels from low to high: under dominant (UDO), recessive (RE), dominant (DO), and over dominant (ODO). To assess the additivity of gene expression in XZM2, a different approach was employed depending on whether the genes in question were parental DEGs (**Supplementary Figure 1**) (Chen, 2013; Zhou et al., 2019).

**Figure 3:**
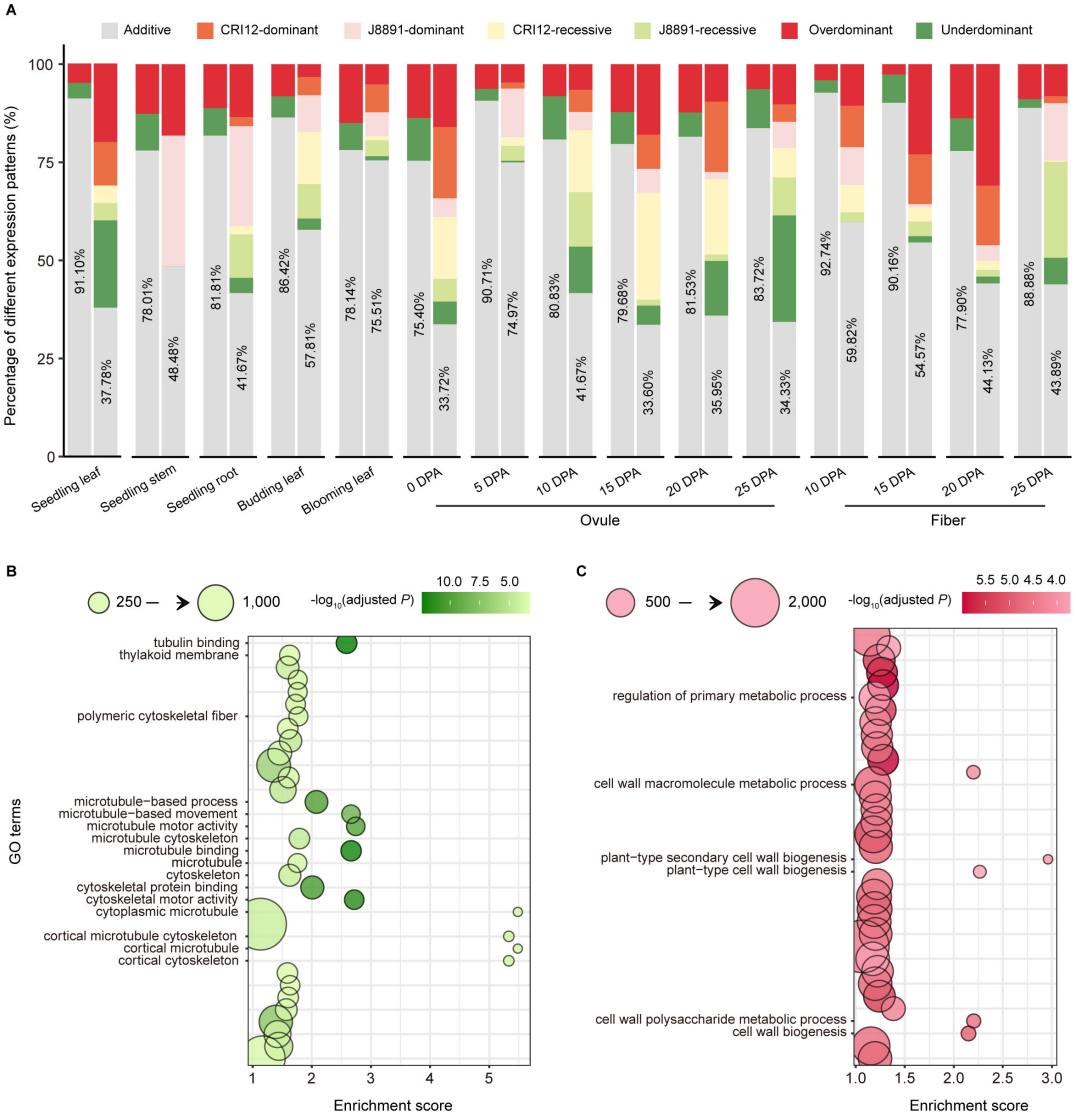
Additive and non-additive gene expression patterns in the hybrid. **(A)** Expression patterns of DEGs and non-DEGs in 15 tissues. The x-axis indicates different tissues, and the y-axis indicates the proportions of different expression patterns. In the paired column chart, expression patterns of non-DEGs are presented on the left, those of DEGs on the right. Colors indicate different expression patterns (gray, additive; dark red, over dominant; dark green, under dominant; orange, CRI12-dominant; pink, J8891-dominant; yellow, CRI12-recessive; light green, J8891-recessive). **(B, C)** GO enrichment results for over dominant genes in seedling leaves **(B)** or ovules at 15 DPA **(C)**. The x-axis indicates enrichment score, and the y-axis the functional terms. Circle size indicates the number of genes enriched in the given term, and color represents -log_10_(adjusted *P* value). Functional terms of particular note are labeled.

Of non-DEGs, the majority exhibited additive expression patterns across tissues, ranging from 80.06% to 94.73% with an average of 87.39%. Only a small fraction of these genes showed expression levels that exceeded the parental range (**Figure 3A**; **Supplementary Table 11**). Among parental DEGs, additive expression was similarly prevalent: in ten out of 15 tissues, more than half (> 50%) of DEGs displayed additive patterns, with the highest proportion found in 5-DPA ovules at 81.40%. In the remaining five tissues, a relatively larger proportion of DEGs (53.33%–59.27%) exhibited non-additive patterns (**Figure 3A**; **Supplementary Table 12**). The most common forms of non-additive expression were DO and ODO patterns, with average proportions of 14.82% and 10.88% respectively (**Supplementary Table 12**). Overall, compared to non-DEGs, DEGs showed a greater tendency towards non-additive expression patterns.

When genes exhibit DO or ODO patterns in a hybrid, their expression levels are maintained at or above the highest level of the parents (**Supplementary Figure 1**). The abundance of DO/ODO genes in XZM2 might play an important role in promoting heterosis. To investigate this possibility, we first examined the expression patterns of SPEGs in the hybrid. Of the total 1,491 SPE events, additive, RE, and DO patterns were exhibited in the hybrid in 47.75% (712), 17.10% (255), and 6.98% (104) cases respectively, with ODO expression occurring at a smaller proportion of 3.29% (49). Thus, the majority (58.02%) of SPE genes in XZM2 are expressed at levels equal to or exceeding the higher parental values, resulting in the activation of an additional 689 genes in the 15 tissues (**Supplementary Figure 2**; **Supplementary Table 13**).

Additionally, previous studies have confirmed that non-additive genes for which hybrid expression levels exceed the higher parent can directly contribute to formation of heterotic performance (Liu et al., 2022). Consequently, Gene Ontology (GO) enrichment analysis was performed to assess the potential biological functions of all genes showing DO and ODO expression patterns. This analysis revealed genes with DO/ODO patterns in seedling stems to be were enriched in terms such as “cytoskeletal motor activity” (GO: 0003774), “microtubule-based movement” (GO: 0007018), and “photosynthetic membrane” (GO: 0034357) (**Figure 3B**; **Supplementary Table 14**). The first two are likely associated with cell elongation, while the last is related to photosynthesis. Meanwhile, in seedling roots, DO and ODO genes were significantly enriched in terms linked to material transport, such as “vesicle-mediated transport” (GO: 0016192) and “transport vesicle” (GO: 0030133) (**Supplementary Table 15**). In ovules at 15 DPA, genes with DO or ODO patterns were enriched in terms that may pertain to fiber development, including “cell wall biogenesis” (GO: 0042546), “cell wall polysaccharide metabolic process” (GO: 0010383), and “plant-type secondary cell wall biogenesis” (GO: 0009834) (**Figure 3C**; **Supplementary Table 16**). These findings not only revealed the diverse biological functions of highly expressed genes in hybrids at specific developmental stages but also implied these genes to have significant roles in the manifestation of heterosis.

### Network hub genes involved in fiber development are highly expressed in the hybrid XZM2

**Figure 4:**
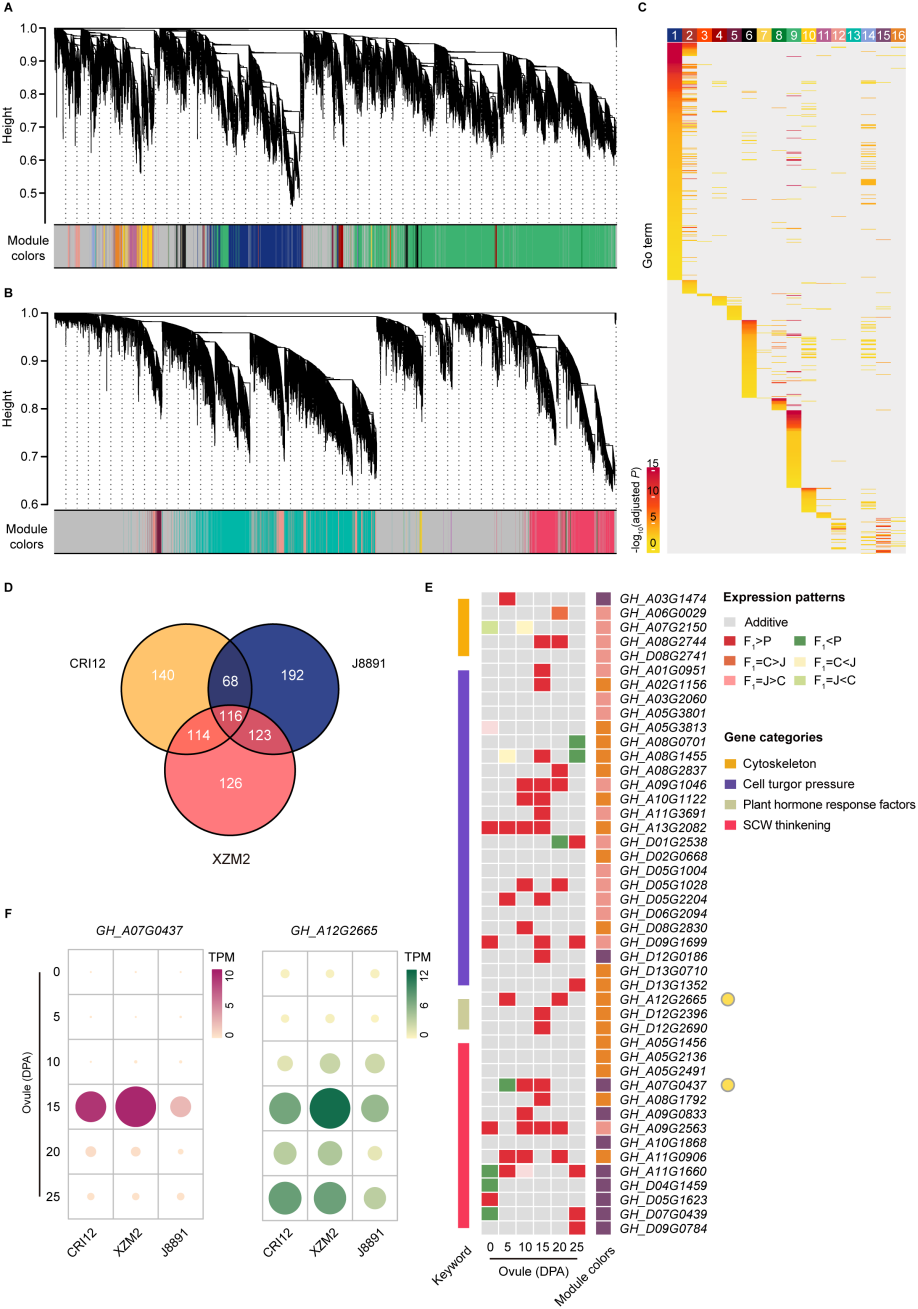
Gene coexpression networks between the hybrid and parents underlying ovules and fibers. **(A, B)** Hierarchical cluster dendrograms showing consensus gene coexpression modules (CMs) within the networks of ovules **(A)** and fibers **(B)** among the two parental lines (CRI12, J8891) and the hybrid (XZM2). Each leaf represents one gene, and each module below the dendrogram is labeled with one color. Genes not coexpressed in all three genotypes are marked in gray. **(C)** GO enrichment of genes in each CM of the ovule network. Color intensity represents enrichment significance. **(D)** Venn diagram showing shared hub genes of the target CMs between CRI12, J8891, and XZM2. **(E)** Shared hub genes involved in fiber development expressed in dominant or over dominant patterns in the hybrid. In the expression heat map, rows represent hub genes and columns represent ovule development stages. Color indicates expression pattern (gray, additive; red, over dominant; orange, CRI12-dominant; pink, J8891-dominant; dark green, under dominant; yellow, CRI12-recessive; light green, J8891-recessive). Rectangles on the left and right sides of the heat map represent biological function classifications and CMs, respectively. **(F)** Expression levels of representative hub genes involved in fiber development. Data are the mean TPM in each genotype at each stage.

To explore differences in gene regulatory networks between the parents and the hybrid, consensus coexpression networks corresponding to ovule and fiber RNA-seq datasets were constructed using weighted gene coexpression network analysis (WGCNA) (Langfelder and Horvath, 2008). These networks effectively summarized the overarching global gene expression patterns shared by all three genotypes (**Figure 1A**). A total of 50,922 genes actively expressed in ovules were allocated to 16 consensus modules (CMs), representing clusters of coexpressed genes that are consistently regulated across genotypes (**Figure 4A**); the sizes of these modules varied from 56 to 22,789 genes each (**Supplementary Table 17**). Similarly, the consensus network for fibers encompassed 48,424 genes organized into seven CMs, with module size ranging from 131 to 12,824 genes (**Figure 4B**; **Supplementary Table 18**).

To quantitate the expression characteristics of each module, the module eigengene (ME) was defined as the first principal component of the gene expression profile for a given CM and its correlation across developmental stages was determined. This revealed CMs within the consensus networks to exhibit distinct correlation patterns throughout the entire developmental period, implying specific temporal expression features (**Supplementary Figures 3**, **4**). Comparison of correlation patterns further showed that the expression characteristics of most modules remained largely consistent across the different genotypes, indicating conservation of the core regulatory mechanism for ovule and fiber development between parents and hybrid. However, genotype-specific perturbations in module expression features were also identified. For example, in the ovule network, Module 12 showed strong correlation with the 10 DPA stage in the hybrid, weakening by 15 DPA, while the reverse trend was observed in the parents (**Supplementary Figure 3**). Similarly, in the fiber network, Module 7 exhibited strong correlation with the 20 DPA stage in the hybrid, which attenuated by 25 DPA; but no significant change was observed in the parental lines (**Supplementary Figure 4**). These perturbations might be associated with the formation of heterosis (Liu et al., 2021).

Further GO enrichment analysis on each CM provided deeper insights into the biological functions of the individual modules. Interestingly, the CMs not only displayed specific temporal expression characteristics but were also highly associated with distinct biological functions (**Figure 4C**, **Supplementary Figure 5**). In cotton, several gene categories are known to be closely related to fiber elongation, including cytoskeleton genes, genes involved in the transportation of osmoticum to maintain high cell turgor pressure, and genes directly or indirectly involved in cell wall loosening, such as plant hormone response factors. Additionally, sucrose synthase genes, cellulose synthase (CESA) genes, and certain NAC transcription factors play important roles during secondary cell wall (SCW) thickening (Fang, 2018). Accordingly, we considered the biological functions of each CM along with their temporal expression characteristics in order to identify CMs highly correlated with fiber development. This distinguished three modules (Module 12, Module 15, and Module 16) in the ovule consensus network and four modules (Module 1, Module 3, Module 6, and Module 7) in the fiber consensus network, genes in which are actively expressed at relevant developmental stages and have potential biological functions highly aligned with the aforementioned categories (**Supplementary Tables 19**–**25**).

To further narrow the analysis scope and pinpoint key regulators of gene expression and function in fiber development, we identified hub genes within these target modules that have high connectivity to other genes in all three genotypes. In the ovule network, this yielded 438, 499, and 479 hub genes from CRI12, J8891, and XZM2, respectively. Among those, 184 hub genes were shared between the two parental lines, while 353 hub genes in XZM2 were shared with both parents (**Figure 4D**; **Supplementary Table 26**). These genes were presumed to have retained their functional characteristics between the parents and the hybrid. As expected, these hub genes were annotated with GO function terms such as “cation transmembrane transporter activity”, “response to auxin”, and “cellulose synthase (UDP-forming) activity”. Taken together, these findings underscore the important role of the three modules and their hub genes in fiber development.

We further compared the expression profiles of these shared hub genes throughout the entire developmental process across the three genotypes. Interestingly, during the mid-to-late stages of fiber development (10–25 DPA), most shared hub genes exhibited a high-parent dominant or over dominant pattern of expression in XZM2 (**Figure 4E**). That is, expression of these genes in the hybrid were maintained at levels equivalent to or even significantly exceeding those of the high-value parent. A similar phenomenon was observed in the fiber network (**Supplementary Figures 6**, **7**; **Supplementary Table 27**). Some notable examples were a member of the CESA gene family in upland cotton and an auxin responsive protein encoding gene. These genes are dominantly expressed during the SCW thickening process and are proposed to have a role in enhancing cotton lint yield and improving quality (Zhang et al., 2015). Correspondingly, expression of these crucial genes in the 15- or 25-DPA ovules of XZM2 significantly surpassed that of the parents (**Figure 4F**). All told, these findings suggest that upregulation of genes with functions related to fiber elongation and thickening might drive the fiber yield and quality heterosis observed in XZM2.

### Allele-specific expression profiles of the hybrid XZM2

**Figure 5:**
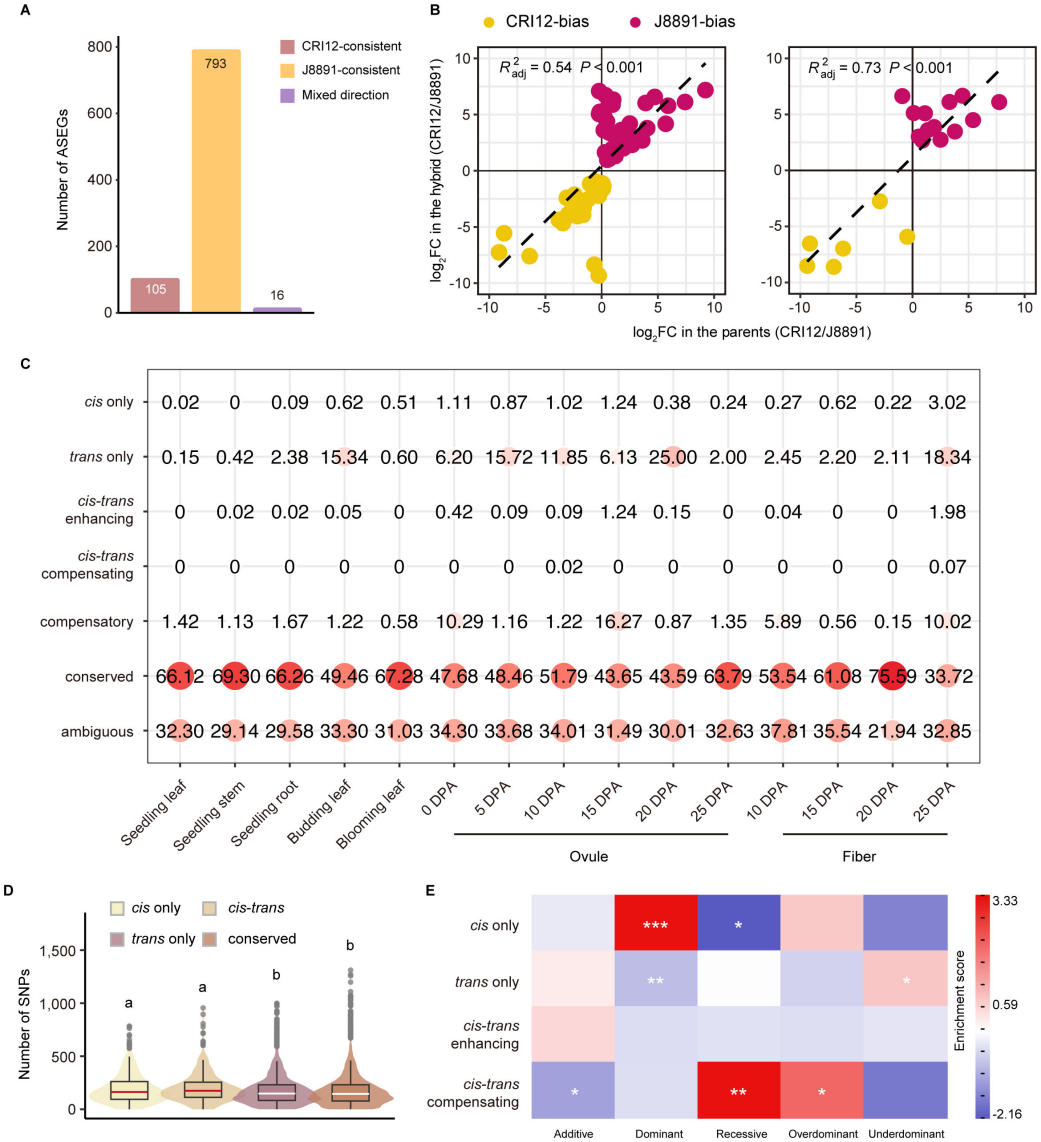
ASE and *cis*/*trans* regulatory variation of genes in the hybrid. **(A)** Classification of ASEGs that show ASE in at least two tissues according to whether the genotype of higher expression changed across tissues (red, CRI12-consistent; yellow, J8891-consistent; purple, mixed direction). **(B)** Correlation between ASE bias in the hybrid and ASEG expression in the parents. The x-axis represents the fold change in ASEG expression between the parents, and the y-axis the fold difference in expression of the parental alleles in the hybrid. The adjusted determination coefficient *R^2^* and *P* value of the linear regression for each tissue are shown for each tissue. **(C)** Classification and identification of allele expression regulation patterns in the parents and the hybrid. Circle size and color represent the proportion of the regulation category in a particular tissue. **(D)** Number of SNPs overlapping gene flanking regions according to gene regulatory pattern. Violin plot colors represent regulatory patterns. Lower case letters above the plot represent significance levels, determined by two-tailed *t*-test. **(E)** Heatmap of gene enrichment and depletion. Rows represent regulation patterns and columns represent expression patterns of genes in the hybrid. Color indicates gene enrichment and depletion magnitude based on residuals of Pearson’s Chisquare test of independence; red indicates a positive residual, where more genes were observed than expected under the null model of independence, and blue indicates fewer genes than expected. Statistical significance was derived from Fisher’s exact test; *P < 0.05, **P < 0.01, ***P < 0.001.

High-confidence single nucleotide polymorphisms (SNPs) in the parental lines CRI12 and J8891 were obtained from high-depth resequencing data, yielding 497,434 SNPs in total (**Supplementary Table 28**). These parental SNPs were then leveraged for the detection of ASE patterns in the hybrid. A total of 1,684 ASE genes (ASEGs) were identified in which the respective parental alleles demonstrated significantly different expression levels (**Supplementary Figure 8**). The expression biases of these ASEGs were found to fluctuate dynamically across tissues, retaining a significant degree of tissue specificity. Indeed, a large proportion of ASEGs (770, 45.72%) exhibited ASE patterns exclusively in a particular tissue. Among the 15 tissues examined, ovules at 15 DPA exhibited the highest number of ASEGs (1,006), representing 59.74% of all identified ASEGs. This was followed by fibers at 25 DPA, in which 767 genes showed ASE (**Supplementary Figure 8**; **Supplementary Table 29**). As tissue differentiation progressed, the directional bias of certain ASEGs changed; however, in contrast to between-parent DEGs, cases of ASE bias reversal were relatively rare. Specifically, only 16 genes (0.95%) exhibited opposing biases across multiple tissues, while a larger subset of ASEGs (898, 53.33%) maintained a consistent parental bias (**Figure 5A**). Notably, the number of genes exhibiting J8891-allele bias (793) significantly outnumbered those biased toward CRI12 alleles (105), and such skew was consistently observed in all tissues (**Figure 5A**; **Supplementary Figure 8**; **Supplementary Table 29**). J8891 is recognized as a high-yielding variety that is well-suited for cultivation in the Yangtze River cotton-growing region, which was also where the parental lines and hybrid were grown and sampled. Based on previous research in rice (Shao et al., 2019), we postulate that the uniform preference for J8891 alleles within XZM2 might be related to environmental factors. This notion highlights the capacity of the hybrid for environmental adaptation and suggests a potential interplay between genetic expression patterns and the ecological context in which a plant is grown.

Interestingly, the allele-level expression difference in most ASEGs was substantial; in 14 of the 15 tissues, it was most common for ASEG alleles to have greater than 8-fold expression difference (**Supplementary Figure 8**). To illuminate the origin of this disparity, we correlated the bias in ASEGs with interparental gene expression differences. This analysis revealed a substantial correlation between most ASE events and differential expression in the parent lines (**Supplementary Figure 9**). Of particular note was the high correlation observed in fibers at 20 DPA (adjusted *R²* = 0.73, *P* < 0.001), which suggests that *cis*-regulation may exert a strong influence on these genes (**Figure 5B**). In contrast, the correlation in ovules at 15 DPA was significantly lower (adjusted *R²* = 0.03), indicating a more pronounced role for *trans*-regulation. These results imply that differential gene expression between the parents was effectively inherited in the hybrid and might contribute to the formation of heterosis.

### *Cis* and *trans* contributions to expression divergence in the hybrid XZM2

We further integrated the analyses of relative expression levels in the parents and ASE ratios in the hybrid in order to infer the source of regulatory variation for 5,501 genes with different alleles (**Supplementary Table 30**). These genes were classified into seven distinct regulatory patterns as previously described (Bao et al., 2019). In line with expectations, some genes (21.94%–37.81%) could not be assigned a definitive regulatory pattern (ambiguous), but the greatest proportion (33.72%–75.59%) maintained expression levels in the hybrid that were not significantly different from levels in the parents (conserved). Contrary to expectations, only a minority of the remaining genes were found to be under the exclusive influence of *cis* effects (*cis*-only, 0.00%–0.51%). A notably higher proportion were only *trans*-regulated (*trans*-only, 0.60%–25.00%), with such genes having pronounced presence in ovules and fibers (**Figure 5C**, **Supplementary Figure 10**). Especially noteworthy was the finding in ovules at 20 DPA, where a quarter of the genes (1,375) were solely subject to *trans-* regulation (**Supplementary Table 30**). These *trans*-regulated genes showed substantial expression differences between the parental lines, but the corresponding alleles in the hybrid exhibited nearly identical expression levels. This underscores the pivotal role of *trans*-regulation in shaping differences between transcriptomes. Genes subject to both *cis*- and *trans*-regulation were relatively rare; however, within those, scenarios where *cis*- and *trans*-regulation exerted opposing effects (0.00%–1.98%) were interestingly more prevalent than scenarios where they were aligned (0.00%–0.07%) (**Figure 5C**, **Supplementary Figure 10**). These findings deviate from previous research conducted in rice and cotton, a discrepancy that may be attributed to species-specific differences and tissue-specific regulatory landscapes (Bao et al., 2019; Shao et al., 2019).

This analysis also revealed that gene regulation patterns are subject to dynamic changes across tissues. Of the 5,501 genes examined, only 294 genes, predominantly within the conserved class, displayed a consistent regulatory pattern. The vast majority of genes (5,207, 94.66%) were under different regulatory controls in different tissues, a dynamic shift that is consistent with the gene expression regulation observed in maize and rice. Moreover, SNPs and insertions/deletion (InDels) were markedly more abundant in *cis*-regulated genes than in *trans*-regulated genes or categories that did not show regulatory divergence, such as conserved and compensatory genes. There was no significant disparity between upstream and downstream regions concerning variant frequency in *cis*-only and *cis*-*trans* regulatory elements (including enhancing and compensating) (**Figure 5D**).

We further performed correlation and enrichment analyses investigating the potential link between gene regulatory patterns and expression modes in the hybrid. Collectively, a significant correlation was observed between regulatory mechanisms and genetic expression patterns (Chi-square test of independence, *P* < 0.05). It is particularly noteworthy that genes with high-parent dominant expression were enriched for *cis*-regulation, while those with low-parent dominance were not, indicating that certain advantageous parental genes may be preserved and enhanced through *cis*-regulation in the hybrid, which could potentially promote heterosis. Additionally, *trans*-regulation was found to be enriched in genes exhibiting under-dominant expression patterns, but notably absent among those having high-parent dominance. Finally, over dominant patterns were significantly represented among genes demonstrating compensatory regulation, a finding that aligns with previous studies on upland cotton. We were unable to identify a significant association with *cis*-*trans* enhancing regulation due to the scarcity of genes subject to that mechanism (**Figure 5E**).

## Discussion

Despite the widespread application of heterosis in agricultural production, which has significantly increased crop yields, understanding of the underlying molecular mechanisms remains limited (Hochholdinger and Baldauf, 2018; Liu et al., 2022). Transcriptome analysis, a key tool in plant biology, has been widely used to map developmental expression profiles and to study gene expression dynamics during plant growth (Ichihashi et al., 2014; Giacomello et al., 2017; Stark et al., 2019; Ryu et al., 2021). However, there is a lack of comprehensive transcriptome data covering multiple genotypes from both parents and hybrids across different growth stages and tissues (Zhou et al., 2019; Liu et al., 2022). This deficit has hindered development of a full understanding of how parental genomic variation shapes the dynamic gene regulatory network in hybrids and thereby contributes to heterosis.

In this study, we constructed dynamic transcriptome profiles for the elite cotton hybrid XZM2 and its parental lines, CRI12 and J8891, covering 15 developmental stages and tissues. This dataset allowed the dissection of parent and hybrid differences in coexpression networks, regulatory patterns, and expression additivity, providing a molecular-level resource with which to explore the potential mechanisms of heterosis.

Previous studies have indicated that differences between parental genomes are the fundamental driver of heterosis (Zhou et al., 2019; Sinha et al., 2020; Xie et al., 2022; Fu et al., 2023). Here, our initial characterization of expression differences also revealed significant interparental disparity, with a total of 25,513 DEGs identified in the 15 tissues, accounting for 52.40% of actively expressed genes. These extensive transcriptional differences appear to play an important role in shaping the heterosis of XZM2. In eight tissues, namely stems and roots at seedling stage, leaves at budding stage, ovules at 5, 10, 15, and 25 DPA, and fibers at 10 DPA, a greater number of genes were expressed at higher levels in J8891. Conversely, the other seven tissues were characterized by more genes with higher expression in CRI12. This suggested that each parent contributed differently to the superior traits of the hybrid through variation in expression activity in particular tissues.

Consistent with that conclusion, the observed transcriptional differences were highly tissue-specific, with the majority of DEGs being differentially expressed in only a limited number of tissues. Of those differentially expressed in multiple tissues (>3), a significant proportion (50.63%) showed a dramatic shift (>4-fold) in the direction of high expression, rather than minor fluctuations. Furthermore, tissue specificity was observed not only in parental DEGs but also in expression additivity and ASE within the hybrid. Genes with ASE showed a lower frequency of bias reversals compared to parental DEGs, but a considerable proportion (45.72%) still maintained significant tissue specificity. Taken together, this variation in gene expression across developmental stages and tissues clearly illustrates the considerable role of transcriptional regulatory diversity.

Given the constitutive role of *cis*-regulation and the presence/absence variation of the gene itself, expression differences of affected genes should be highly conserved across different tissues (Yáñez-Cuna et al., 2013; Schmitz et al., 2022; Zhang et al., 2022). However, *cis*-only regulation was observed in 0.00%–0.51% of genes, a very small fraction. Rather, a high proportion of genes appeared to be regulated by *trans*-acting factors or other complex regulatory mechanisms. In all, our findings regarding expression diversity and dynamics underscore the difficulty in explaining heterotic mechanisms based solely on variations in parental gene expression.

Additionally, when examining expression additivity in the hybrid, we noted that previous studies have shown a higher proportion of non-additive expression pattern (Zhou et al., 2019). These differences may be caused by various factors, including but not limited to species specificity, tissue type, developmental stage specificity, and differences in analytical methods. Variations in gene regulatory networks between different species may lead to differences in non-additive expression patterns. Furthermore, the sensitivity of specific tissues or developmental stages may also affect the detection of non-additive expression. However, there is no significant correlation between the number of non-additive expression genes in hybrids and the level of heterosis. Therefore, identifying key non-additive regulatory factors remains crucial for elucidating the molecular mechanisms of heterosis.

Many studies have explored the disparities in transcriptome profiles between parents and hybrids, yet the utility of synthesizing the expression regulatory networks of all three genotypes has largely been overlooked (Shen et al., 2017; Yuan et al., 2017; Zhou et al., 2019). In revealing the highly conserved coexpression network shared by XZM2 and its two parental lines, the present study provides a unified perspective on gene clusters (modules) and expression patterns across all three genotypes. The network analysis also enabled investigation of whether and how differences in the regulatory network, especially hub genes, contribute to the formation of heterosis. Our approach contrasted with traditional analyses that focus on differential gene expression based on a single time point or tissue, as the network we constructed effectively integrated transcriptional information across the entire developmental period of the ovules and fibers across different genotypes, reducing the potential for false-positive results.

Subsequent identification of network hub genes allowed more precise targeting of key regulatory factors involved in XZM2 ovule and fiber development. Examination of gene expression patterns revealed a large number of hub genes associated with fiber elongation and thickening processes to exhibit high-parent dominant or over dominant expression patterns at mid-to-late developmental stages (10–25 DPA). This was consistent with the phenotypic performance of XZM2 (Li et al., 1997, Li et al., 2001), and suggests that upregulated expression of key development genes directly contributed to heterosis. These findings also provide molecular evidence to support the hypotheses of dominance complementation and overdominance (Jones, 1917; Xiao et al., 1995). Some genes with over dominant patterns in the hybrid showed no interparental difference in expression level, which discrepancy might result from the combined effects of *cis-* and *trans-*regulation. That is, gene expression variation unique to the hybrid may be due to a combinatorial effect of the two parental genomes on regulatory factors. Although we did not observe SPEGs and ASEGs with high-parent dominant or over dominant expression patterns among the hub genes highly related to fiber development, this does not exclude their potential role in heterosis. We believe that these genes may play a role in other critical tissues or developmental stages that were not sampled, or their contribution to heterosis might not have been fully revealed due to the current limitations in gene function annotation.

In summary, this study systematically investigated transcriptional differences between the elite cotton hybrid XZM2 and its parental lines across different developmental stages and tissues, and thereby provides a rich dataset for linking transcriptional differences to heterotic phenotypes. The results deepen our understanding of the roles of gene expression and regulatory network differences in the formation of heterosis.

## Data availability statement

The re-sequencing data for the CRI12 and J8891 are available in the NCBI Sequence Read Archive (SRA) under accession number PRJNA1045850. The Illumina RNA-seq data for J8891, CRI12 and XZM2 are available in NCBI under accession number PRJNA1045879.

## Author contributions

CH: Conceptualization, Data curation, Formal analysis, Funding acquisition, Investigation, Methodology, Project administration, Resources, Software, Supervision, Validation, Visualization, Writing – original draft, Writing – review & editing. YCh: Conceptualization, Data curation, Formal analysis, Funding acquisition, Investigation, Methodology, Project administration, Resources, Software, Supervision, Validation, Visualization, Writing – original draft, Writing – review & editing. YH: Formal analysis, Investigation, Project administration, Writing – review & editing. LF: Formal analysis, Investigation, Methodology, Writing – review & editing. ZS: Formal analysis, Investigation, Project administration, Writing – review & editing. JC: Visualization, Writing – review & editing. YCa: Investigation, Visualization, Writing – review & editing. XG: Project administration, Writing – review & editing. TZ: Project administration, Supervision, Writing – original draft, Writing – review & editing.
