# Supplementary material for: Dynamic patterns of gene expressional and regulatory variations in cotton heterosis
Source: Front Plant Sci. 2024 Aug 6;15:1450963. doi: 10.3389/fpls.2024.1450963 (PMC11333441; doi:10.3389/fpls.2024.1450963)
Supplement: Supplementary file 1 [file DataSheet_1.docx]

# Supplementary figures


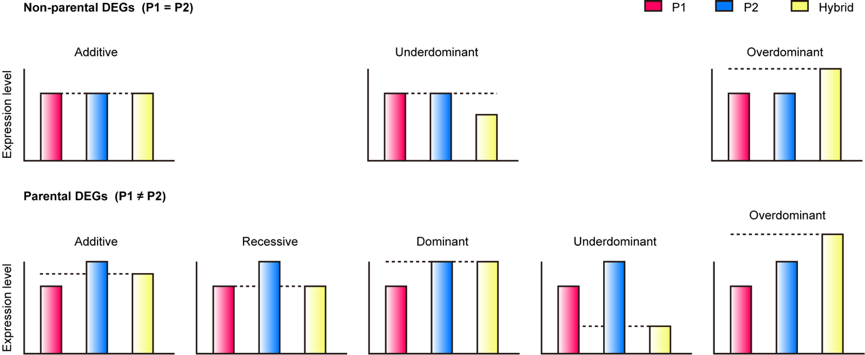


**Supplementary Figure 1. Expression patterns of parental DEGs and non-DEGs in the hybrid.**

The x-axis represents different genotypes (red and blue, the parents; yellow, the hybrid), and the y-axis represents the relative expression levels of genes. Non-parental differentially expressed genes (DEGs) exhibit expression patterns in the hybrid that increase from low to high as underdominant, additive, and overdominant. Similarly, parental DEGs exhibit expression patterns in the hybrid that increase from low to high as underdominant, recessive, additive, dominant, and overdominant.


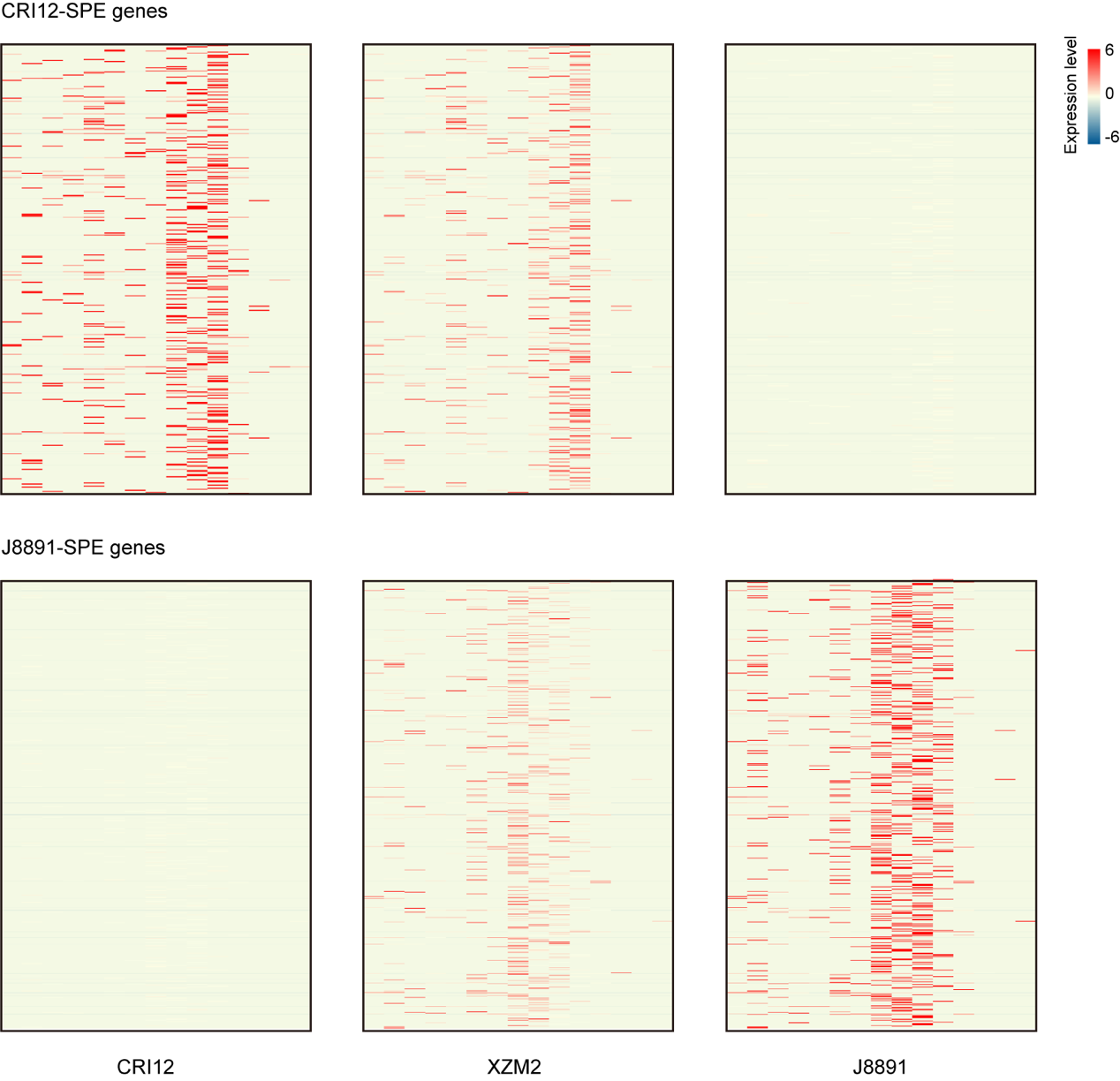


**Supplementary Figure 2. Heat map of the expression levels of SPEGs in the parents and the hybrid.**

The three heat maps in the first and second rows represent the expression of CRI12-SPE genes and J8891-SPE genes in different genotypes, respectively. In each heat map, columns represent different tissues, and rows represent different SPEGs. The color of the cells indicates the relative expression levels of the SPEGs.


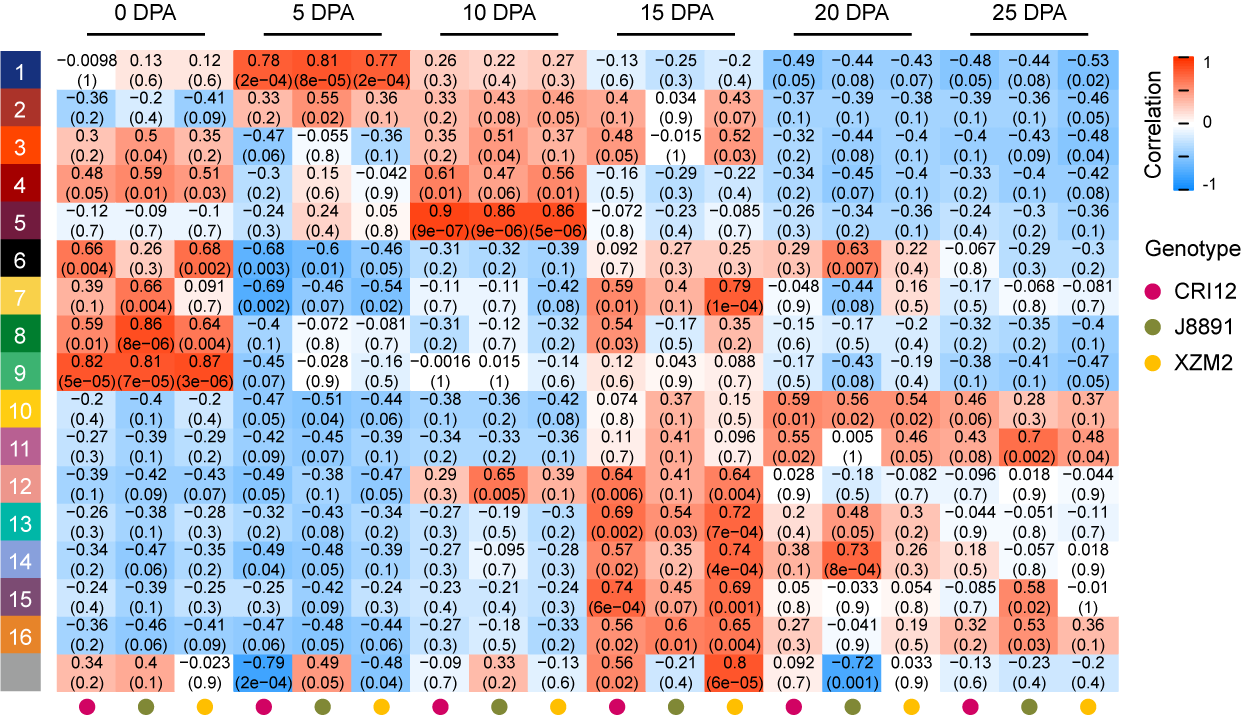


**Supplementary Figure 3. Heat map of the correlation between CMs and developmental stages in the ovule consensus coexpression network.**

Rows represent different CMs. Columns represent different genotypes and tissues. The color of the cells represents correlation (red, positive correlation; blue, negative correlation). The degree of correlation and *P* values are indicated in the plot.


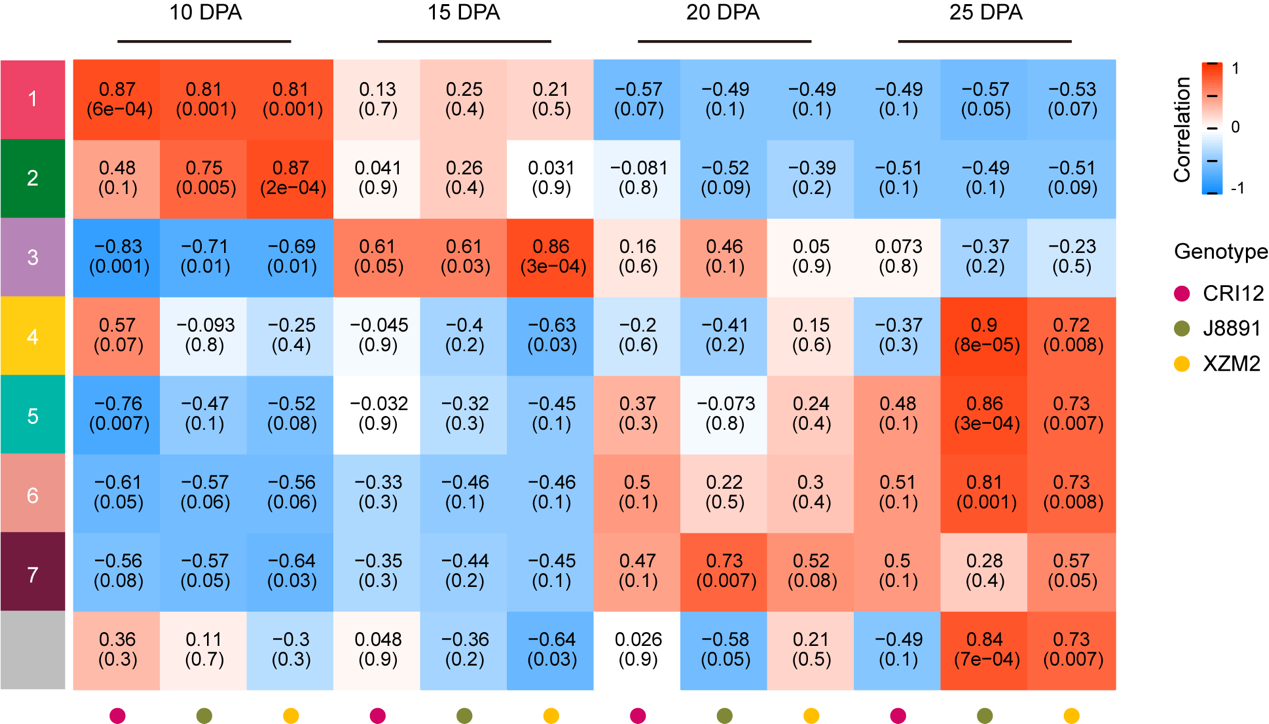


**Supplementary Figure 4. Heat map of the correlation between CMs and developmental stages in the fiber consensus coexpression network.**

Rows represent different CMs. Columns represent different genotypes and tissues. The color of the cells represents correlation (red, positive correlation; blue, negative correlation). The degree of correlation and *P* values are indicated in the plot.


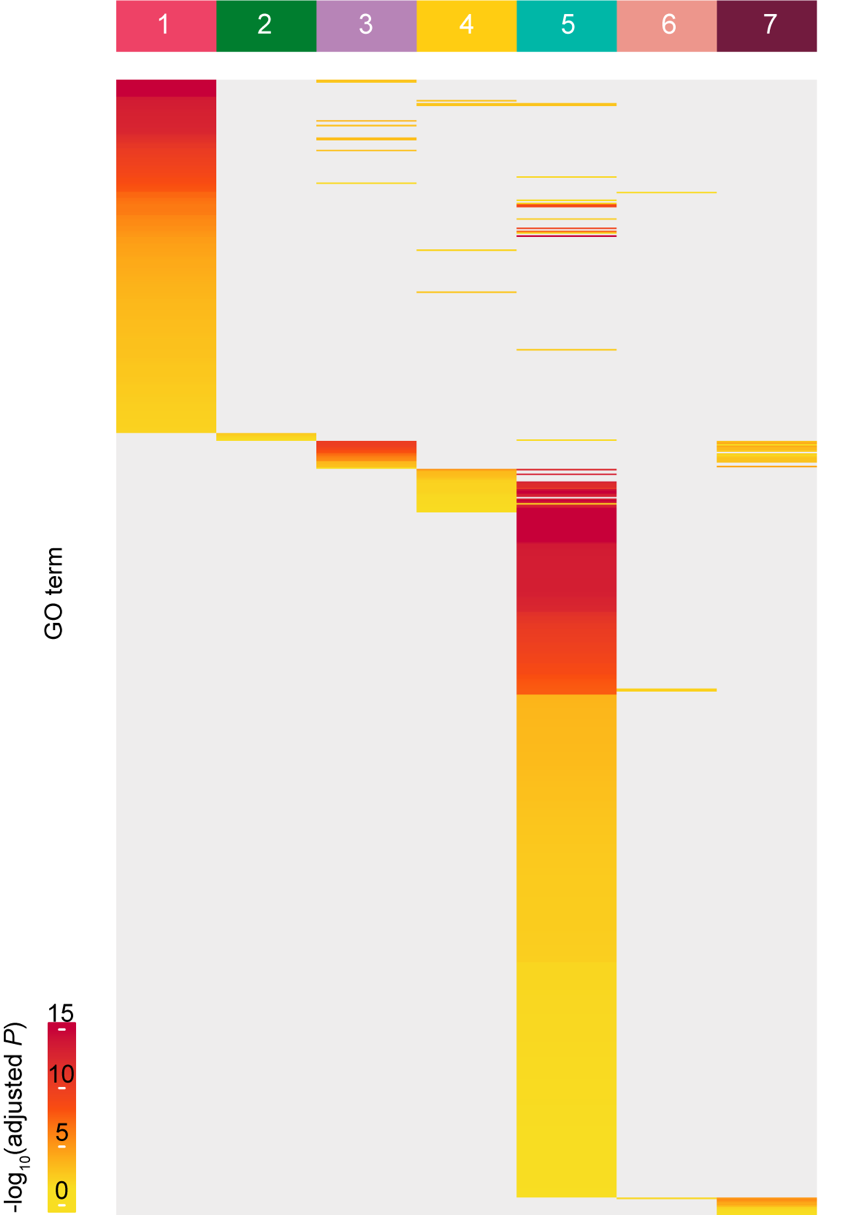


**Supplementary Figure 5. GO enrichment of genes in each CM of the fiber network.**

Rows represent different GO terms. Columns represent different CMs. Color intensity represents the significance of GO term enrichment.
