# Supplementary material for: Dynamic patterns of gene expressional and regulatory variations in cotton heterosis
Source: Front Plant Sci. 2024 Aug 6;15:1450963. doi: 10.3389/fpls.2024.1450963 (PMC11333441; doi:10.3389/fpls.2024.1450963)
Supplement: Supplementary file 2 [file DataSheet_2.docx]

# **Supplementary figures**


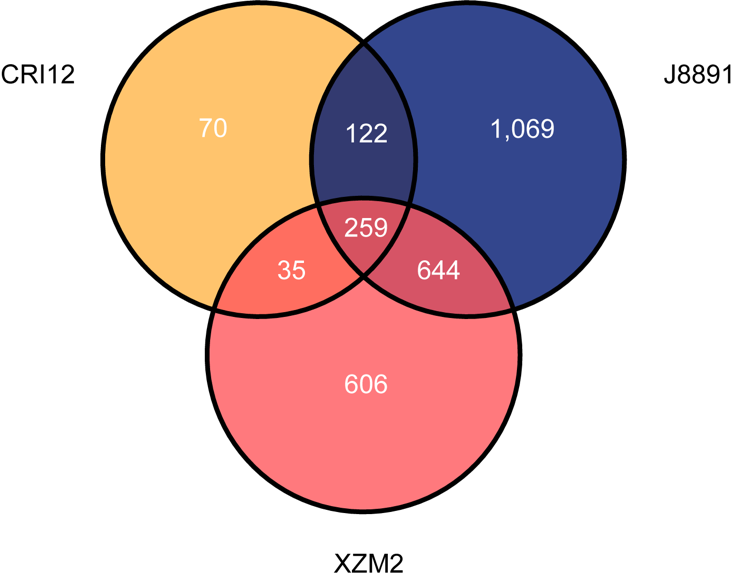


**Supplementary Figure 6. Venn diagram showing the shared hub genes of the target CMs in the fiber network between CRI12, J8891, and XZM2.**

The color of circles indicates different genotypes (yellow, CRI12; blue, J8891 and red, XZM2).

**
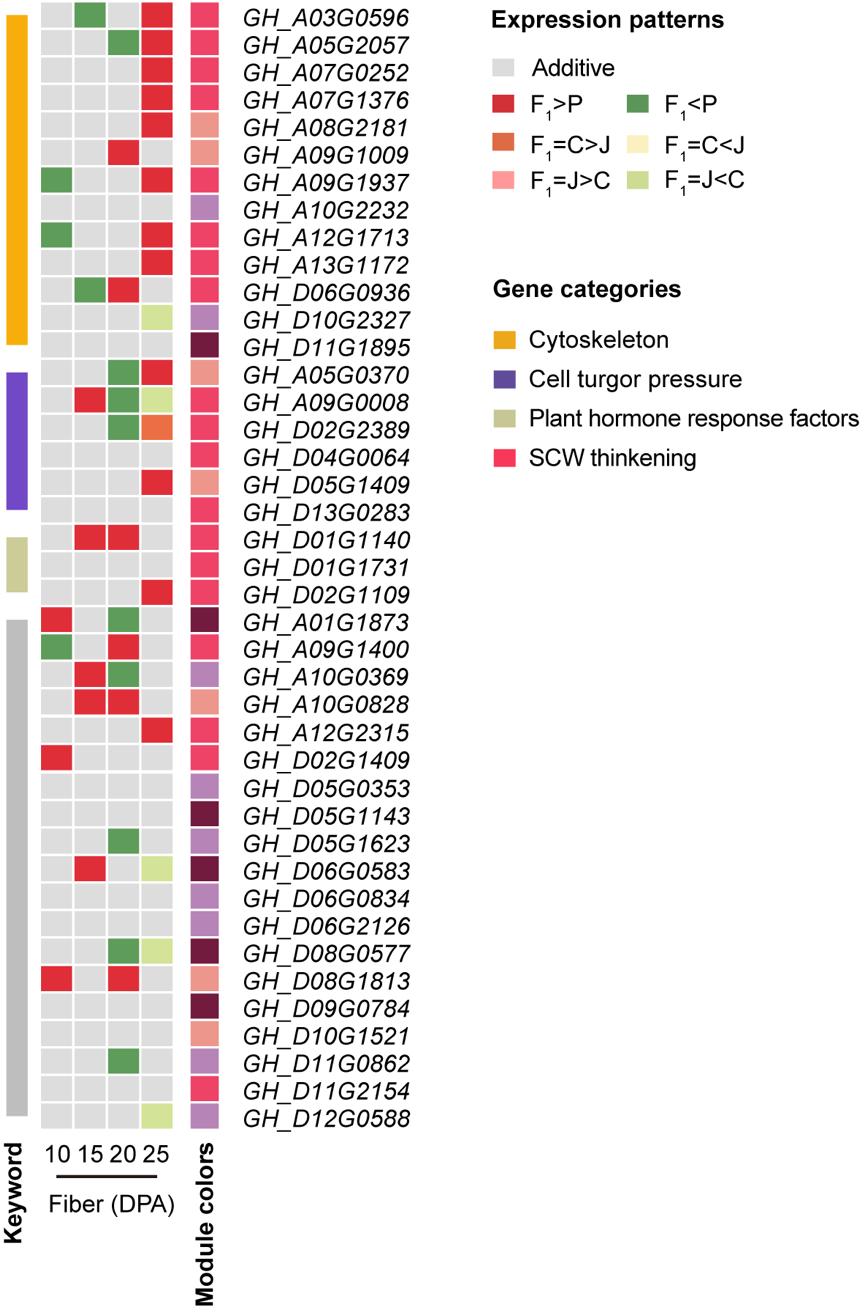
**

**Supplementary Figure 7. Shared hub genes involved in fiber development expressed in dominant or overdominant patterns in the hybrid.**

In the expression heat map, rows represent hub genes and columns represent fiber development stages. The color of the cell indicates the expression pattern of the gene (gray, additive; red, overdominant; orange, CRI12-dominant; pink, J8891-dominant; dark green, underdominant; yellow, CRI12-recessive; light green, J8891-recessive). The rectangles on the left and right sides of the heat map represent the biological function classification and CMs of the gene, respectively.

**
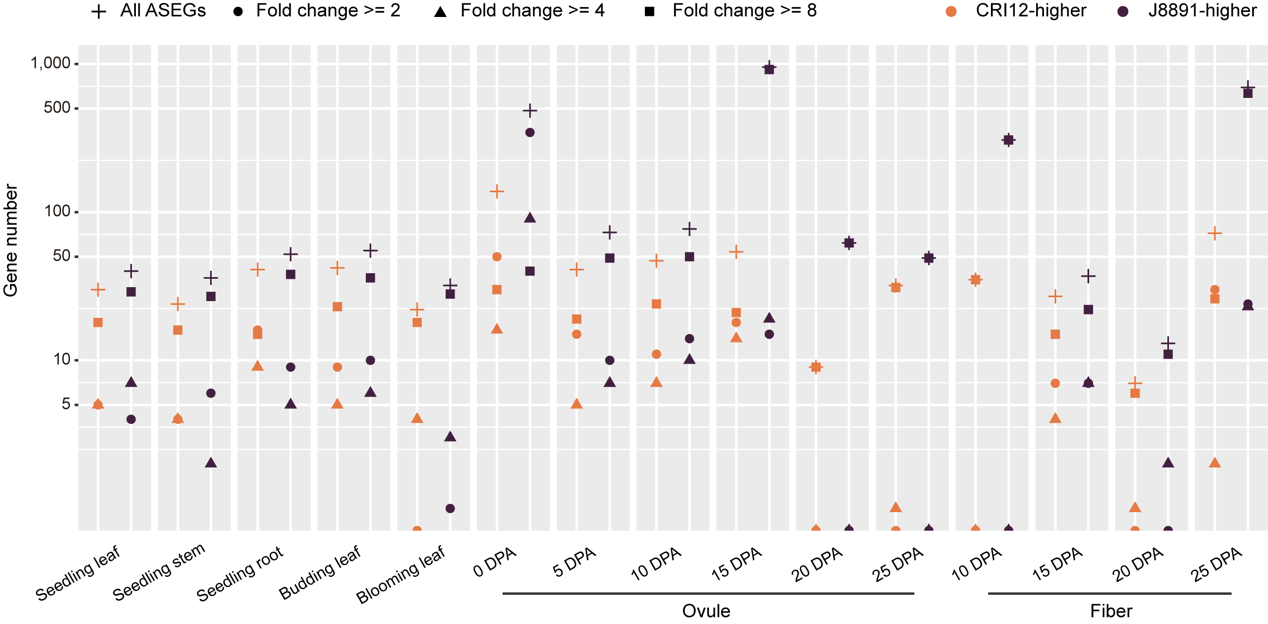
**

**Supplementary Figure 8. Number of ASEGs in 15 tissues.**

The number of ASEGs for each tissue is indicated by the cross symbols, with the genotype exhibiting higher expression indicated by color (purple, CRI12; yellow, J8891). Subsets of genes with >= 2-fold, >= 4-fold, or >= 8-fold changes in expression are also indicated by symbols.

**
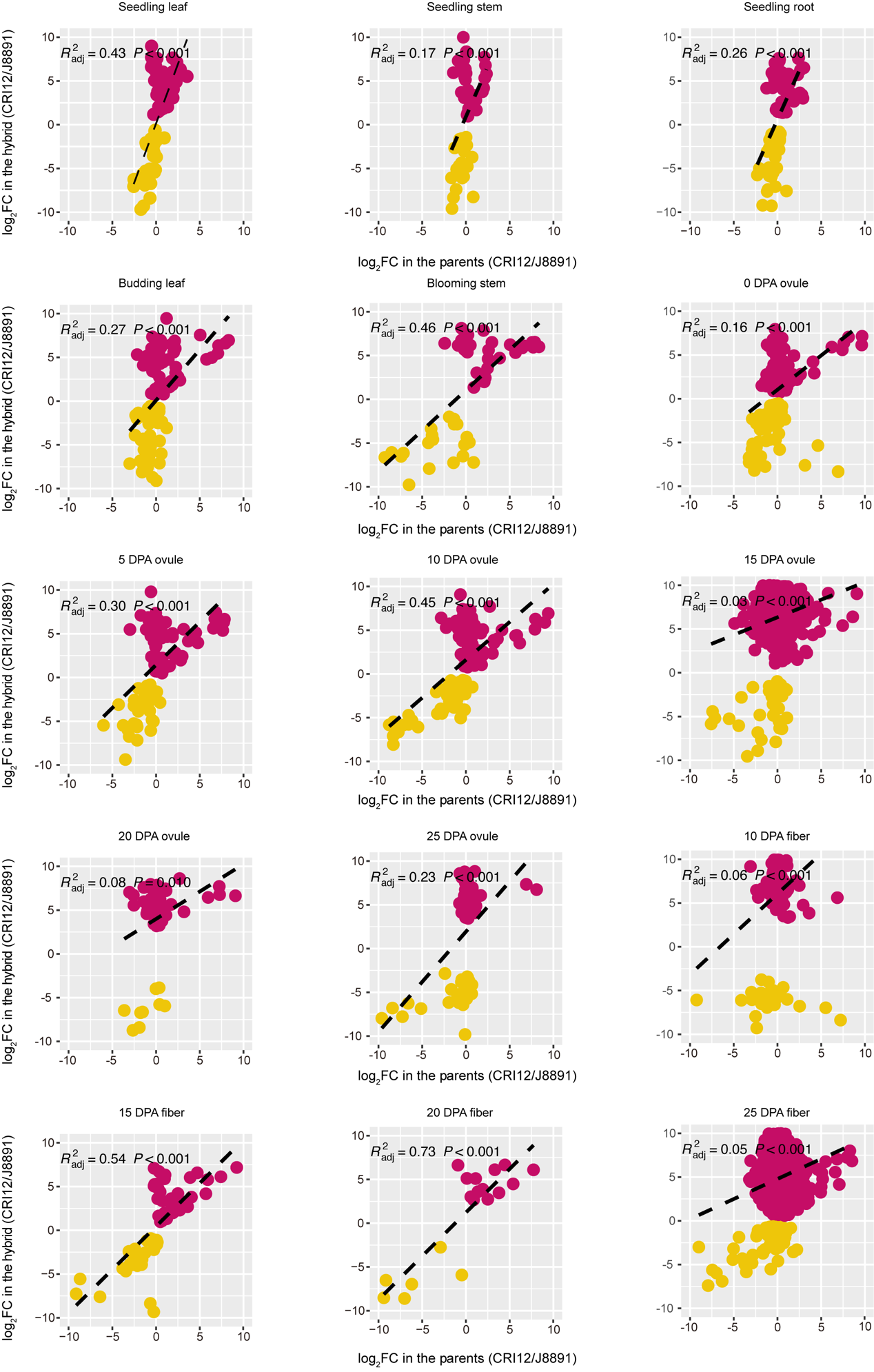
**

**Supplementary Figure 9. Correlation between the ASE bias in the hybrid and the expression of ASEGs in the parents.**

The x-axis represents the fold change in expression of ASEGs between the parents, and the y-axis represents the fold difference in expression of the two parental alleles in the hybrid. The adjusted determination coefficient *R^2^* and *P* value of the linear regression for each tissue.

**
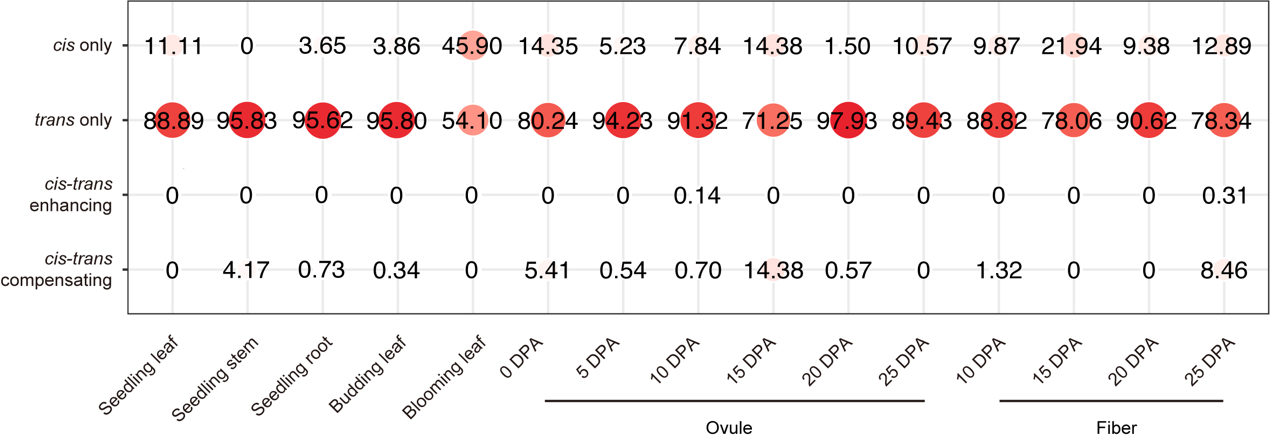
**

**Supplementary Figure 10. Classification and identification of expression regulation patterns (A ≠ 0) of alleles in the parents and the hybrid.**

The size and color of the circles represent the proportion of the regulation category in a particular tissue.
